# Rosuvastatin attenuates contrast-induced nephropathy through modulation of nitric oxide, inflammatory responses, oxidative stress and apoptosis in diabetic male rats

**DOI:** 10.1186/s12967-015-0416-1

**Published:** 2015-02-12

**Authors:** Jie Deng, Guijun Wu, Chen Yang, Yi Li, Quanmin Jing, Yaling Han

**Affiliations:** Department of Cardiology, the General Hospital of Shenyang Military Region, Chinese People’s Liberation Army, Shenyang, Liaoing China

**Keywords:** Diabetic, Contrast-induced nephropathy (CIN), Rosuvastatin

## Abstract

**Background:**

Contrast-induced nephropathy (CIN) is an important cause of acute renal failure. We observe the effect of rosuvastatin on preventing CIN in diabetic rats in current study.

**Methods:**

Diabetic rats were then divided into five groups: 1 diabetic rats (D), 2 diabetic rats + contrast media (DCM), 3 diabetic rats + rosuvastatin (DR), 4 diabetic rats + contrast media + rosuvastatin (DRCM), 5 non-diabetic rat control (NDCM). Contrast-induced nephropathy was induced by intravenous injection a single dose of indomethacin (10 mg/kg), double doses of N-nitro-L-arginine methyl ester (10 mg/kg) and a single dose of high-osmolar contrast medium meglumine amidotrizoate (6 ml/kg). DR and DRCM group rats were treated with rosuvastatin (10 mg/kg/day) by gavage for 5 days. At the end of treatment, the experimental groups were sacrificed, and their renal tissues were investigated histopathologically beside assessments of functional activities, nitric oxide metabolites, and oxidative stress and apoptic markers.

**Results:**

After 6 days, serum creatinine and urine microprotein were increased, and creatinine clearance, kidney nitrite were decreased in DCM rats compared with NDCM, D, DR and DRCM groups. Histopathology scores in group DCM were increased compared with groups NDCM, D and DR, but lower in group DRCM than in group DCM (p < 0.01). Kidney thiobarbituric acid-reacting substances (TBARS), serum malondialdehyde (MDA), and serum protein carbonyl content (PCC) were increased, and serum thiol was decreased in the DCM group compared with groups NDCM, D and DR; however, these results were reversed in group DRCM compared with group DCM. Both expression of IL-6, TNF-α and the percentage of apoptotic cells were increased in group DCM than in groups NDCM, D and DR, but they were decreased in group DRCM than in group DCM. The expression of phospho-p38, cleaved capase-3, and the Bax/Bcl-2 ratio, were increased in group DCM than in groups NDCM, D and DR, but were decreased in group DRCM than in group DCM.

**Conclusions:**

Our study demonstrated that rosuvastatin treatment attenuated both inflammatory processes and apoptosis and inhibited oxidative stress and the p38 MAPK pathway in a diabetic rat model in the setting of CIN.

## Introduction

Contrast-induced nephropathy (CIN) is one of important cause of hospital-acquired acute renal failure [1], which is associated with an increase of 25% or more, or an absolute increase of 0.5 mg/dl or more in serum creatinine from baseline value, at 48–72 h following the exposure to contrast media. The incidence of CIN is recognized as a significant contributor to morbidity and mortality in patients receiving coronary angiography and subsequent intervention [2,3]. Diabetes mellitus (DM) is an important risk factors for CIN [4]. In combination with other risk factors, DM aggravates the situation. CIN develops more frequently after administration of contrast media in DM patients than in patients without diabetes [5]. Additionally, diabetic patients with CIN have a significantly decreased survival rate compared with nondiabetic patients [5]. The exact mechanism of CIN has not been cleanly understand. Renal hypoxia, combined with the generation of reactive oxygen species, plays a central role in the pathogenesis of CIN, and the diabetic kidney is particularly susceptible to intensified hypoxic and oxidative stress following the administration of contrast media [6]. The pathophysiology of this vulnerability is complex and involves various mechanisms, including a priori enhanced tubular transport activity, oxygen consumption, and the generation of reactive oxygen species [7].

Dyslipidemia is common in diabetic patients. Typical dyslipidemia of diabetic patients is hypertriglyceridemia, low high-density lipoprotein cholesterol (HDL-C) levels, high small and dense low-density lipoprotein (LDL) particles levels [8]. Most of diabetic patients have complications such as retinopathy, nephropathy, and neuropathy. Nearly 40% of diabetic patients have the risk of developing diabetic nephropathy [9]. Diabetic nephropathy is characterized by an increased in albuminuria and a decreased in creatinine clearance. Statins are 3-hydroxy-3-methylglutaryl coenzyme A reductase inhibitors effective reduce both total and LDL cholesterol. Studies have found unequivocally that lowering LDL-C, particularly with statins, decreases the risk of cardiovascular deaths and events [10]. Recently years, there has been increasing interest in the role of statins in decreasing CIN risk in patients receiving contrast media [11-13]. This is owing to both their ability to anti-inflammatory and improve endothelial function properties, and their anti-apoptotic and anti-oxidant effects [14-16]. Rosuvastatin is a new generation of statin [14], which reduces levels of circulating LDL-C. In addition to its beneficial cholesterol lowering effects, rosuvastatin has been shown to protect against inflammatory, oxidant, and thrombotic effects, and may improve endothelial function. These effects of rosuvastatin have been translated into beneficial effects on atherosclerosis and have resulted in a significant reduction in cardiovascular events [17]. Our recent study reports that rosuvastatin reduces the risk of CIN in DM patients [18], and Leoncini et al. show that patients with renal dysfunction benefited from rosuvastatin therapy [19]. However, the mechanisms of rosuvastatin’s reduction of CIN have not been reported. Furthermore, there is no evidence that rosuvastatin simultaneously attenuates inflammation, apoptosis or oxidation in vivo, each of which is prominent in CIN in the setting of diabetes mellitus. Therefore, the aim of the current study was to evaluate the anti-inflammatory, anti-apoptotic and anti-oxidant effects of rosuvastatin on CIN in diabetic rats.

## Material and methods

### Animals

Male Wistar rats (350–400 g) obtained from the Central Animal House of the General Hospital of Shenyang Military Region. Ethics was given by the Ethics Committee of the General Hospital of Shenyang Military Region. The experimental and animal care procedures were approved by the Institutional Animal Care and Use Committee of the General Hospital of Shenyang Military Region. The investigation conformed to the Guide for the Care and Use of Laboratory Animals published by the US National Institutes of Health.

### Induction of diabetes

After 12 h of fasting, streptozotocin (60 mg/kg,Sigma, St. Louis, MO) was injected into the tail vein. After 24 h, blood glucose levels over 250 mg/dl were considered diabetic. Diabetic state was confirmed by testing blood glucose levels on a daily basis.

### Rosuvatatin-treatment

Rosuvastatin was orally administered at a dose of 10 mg/kg/day by oral gavage for 5 consecutive days (days 1 to 5). The dose of rosuvastatin was decided according to Bae et al. [20].

### Induction of nephropathy

CIN was induced by a single intravenous injection of indomethacin (10 mg/kg), double doses of N-nitro-L-arginine methyl ester (10 mg/kg, twice at 15 and 30 min) and a single dose of meglumine amidotrizoate 60% (6 ml/kg; Urovist-Angiographin, Schering AG, Germany), according to Kedrah et al. [21].

### Animal grouping and experimental work

Thirty-two male diabetic rats were allocated randomly to the following 4 equal groups (n = 8/group): diabetic group (D), diabetic + contrast media (DCM), diabetic + rosuvastatin (DR), diabetic + rosuvastatin + contrast media (DRCM). Another 8 healthy male rats + contrast media served as the non-diabetic rat control (NDCM). All the experimental groups were allowed to recover for 24 h in metabolic cages before being sacrificed on the day 6. Rats were sacrificed by light ethyl ether anesthesia. Blood was collected by heart puncture, and we separated serum for renal function testing. The kidneys were removed for both biochemical and histopathological investigations.

### Determination of creatinine in serum and urine

Serum samples were analyzed for biochemical parameters within 24 h. Urine samples were also collected and analyzed. Concentrations of creatinine and urea in both serum and urine were analyzed as indicators of injured glomerular function. Creatinine clearance (ml/min) was calculated according to the formula: UV/P (U = creatinine concentration in urine (mg/dl), V = urine volume per min (ml/min) and P = serum creatinine (mg/dl)). Fractional excretion of sodium (FENa) was calculated by formula (urine sodium/serum sodium) × (serum creatinine/urine creatinine) × 100.

### Inflammatory parameters

Serum concentrations of interleukin-6 (IL-6) and tumor necrosis factor-alpha (TNF-ɑ) were analyzed by enzyme-linked immunosorbent assay (ELISA) kits (R&D Systems, USA) according to the manufacturer’s instructions.

### Determination of biomarkers of oxidative stress

The thiobarbiturates (TBARS) were determined according to Toprak et al. [22]. Levels of malondialdehyde (MDA) were measured using a spectrophotometric method. Sample absorbance was assayed at 532 nm and calculated by the absorbance of a standard. Thiol levels were measured using a method based on the thiol-disulphide interchange reaction between thiols and 5, 5’-dithio-bis-(2-nitrobenzoic acid). The serum protein carbonyl content (PCC) were analyzed according to Reznick AZ al.[23].

### Measurement levels of total nitrite/nitrate

Nitrite and nitrate, the stable metabolic products of kidney tissues nitric oxide (NO), were measured by the Griess reaction for the simultaneous evaluation of nitrite and nitrate concentrations [24].

### Western blotting

The renal tissue was homogenized in lysis buffer (pH 7.5, containing 300 mM NaCl, 50 mM Tris-Cl, 0.5% Triton X-100, protease-inhibitor) and incubated at 4°C for 30 min. The lysates were centrifuged at 15,000 rpm for 20 min at 4°C. Concentrations of protein were determined by Bradford protein assay reagent (Bio-Rad, USA), and 30 g proteins were loaded onto 10% SDS/PAGE. The gels were transferred to a nitrocellulose membrane and reacted with each antibody. Antibodies against phospho-p38, total-p38, cleaved capase-3 and total caspase-3 were obtained from Santa Cruz Biotechnology. Antibodies against β-actin, Bax, and Bcl-2 were obtained from Cell Signaling Technology. The ECL Western Blotting System was obtained from Amersham Biosciences.

### Histopathological investigation

Kidneys were removed for histological analysis under a light microscope. Renal specimens from the used experimental groups were removed and immediately fixed in 10% phosphate buffered formalin (pH 7.4) for 24 hours, followed by dehydrating in ascending grades of ethyl alcohol, cleared in xylene and mounting in molting paraplast 58-62C°. Five μm thick were cut and stained with hematoxylin/eosin. The criteria of injury and necrosis were analyzed semi-quantitatively method according to Ahmad [25]. Briefly, method of pathological score were as follows: 0 = no damage; 1 = minimal injury; 2 = moderate injury; 3 = intermediate injury; 4 = severe injury; 5 = very severe injury.

### TUNEL assay

The assay was determine by a TUNEL detection kit by the manufacturer's instructions (Cell Signaling Technology, USA). Briefly, kidney samples were cut to 5 μm sections and incubated with 15 μg/ml proteinase K for 10 min and then washed with PBS. Sections were immersed in terminal deoxynucleotidyltransferase and biotinylated dUTP in TdT buffer for 90 min. The sections were incubated with anti-horseradish peroxidase-conjugated antibody at room temperature for 30 min, and the resulting signals were visualized with diaminobenzidine. TUNEL-positive tubular cell numbers were counted at random in 20 non-overlapping cortical fields under 200 × magnification.

### Statistical analysis

Data are expressed as mean ± SD. Analyses were performed with SPSS 13.0. Group comparisons were evaluated by ANOVA followed by Tukey’s test. Within-subject comparisons of continuous variables were carried out by a paired *t*-test. P value of <0.05 was considered statistically significant.

## Results

### Biochemical parameters

Levels of serum creatinine were notably increased in the DCM group compared with the NDCM, D or DR groups on day 6 (P < 0.01). Treatment with rosuvastatin significantly decreased serum creatinine levels (DRCM vs DCM, P < 0.01) (Table 1), indicative of rosuvastatin protection against CIN in this model. On day 6, creatinine clearance was notably lower in group DCM compare with groups NDCM, D, and DR (P < 0.01). Levels were increased notably in group DRCM than in group DCM (P < 0.01). There were no differences in either levels of BUN or FENa among the groups. Levels of urine microprotein were notably higher in group DCM compare with groups NDCM, D, DR and DRCM On day 6 (P < 0.01). Additionally, levels of Urine microprotein were notably higher in group NDCM than in groups D and DR (P < 0.05) (Table 1).

**Table 1:** **Renal functional parameters in the study groups** (**mean** ± **SD)**

	**NDCM (n = 8)**	**D (n = 8)**	**DR (n = 8)**	**DCM (n = 8)**	**DRCM (n = 8)**
Serum creatinine (mg/dl)	0.56 ± 0.05#	0.36 ± 0.03	0.38 ± 0.03	0.89 ± 0.06*	0.42 ± 0.04+
Serum BUN (mg/dl)	14.98 ± 1.62	15.23 ± 1.32	16.26 ± 1.48	16.38 ± 1.93	16.45 ± 1.53
Creatinine clearance (ml/min)	1.53 ± 0.18#	2.68 ± 0.25	2.59 ± 0.24	1.08 ± 0.09*	1.98 ± 0.22+
FENa (%)	0.32 ± 0.15	0.29 ± 0.10	0.28 ± 0.09	0.31 ± 0.14	0.33 ± 0.13
Urine microprotein (mg/dl)	70.2 ± 22.1#	45.2 ± 11.3	38.6 ± 14.3	108.5 ± 23.4*	69.3 ± 18.2#

NDCM: non-diabetic rats + contrast media, D: diabetic group, DCM: diabetic rats treat + contrast media, DR: diabetic rats + rosuvastatin, DRCM: diabetic rats + rosuvastatin + contrast media.

*P < 0.01 vs NDCM, D, D R and DRCM; + P < 0.05 vs NDCM, D and DR, # P < 0.05 vs D, DR.

### Renal nitric oxide production

From Table 2, kidney nitrite levels was notably decreased in the DCM group than in group D (P < 0.01). Additionally, levels of kidney nitrite in group DCM were notably decreased compared with those in groups NDCM, DR and DRCM (P <0.01). Levels of kidney nitrite in DRCM were increased than those in NDCM (P < 0.01). Additionally, levels of the kidney nitrite in group NDCM were lower compared with groups D and DR (P < 0.05).

**Table 2:** **Nitric oxide and nitrite/nitrate levels (nmol/mg protein) in the kidneys (mean ± SD)**

	**NDCM (n = 8)**	**D (n = 8)**	**DR (n = 8)**	**DCM (n = 8)**	**DRCM (n = 8)**
Nitrite	0.038 ± 0.003#	0.049 ± 0.006	0.052 ± 0.007	0.026 ± 0.002*	0.048 ± 0.004**
Nitrate	6.543 ± 0.721	6.383 ± 0.691	6.430 ± 0.683	6.625 ± 0.822	6.831 ± 0.760
Total nitrite/nitrate	6.581 ± 0.702	6.432 ± 0.721	6.482 ± 0.694	6.651 ± 0.831	6.879 ± 0.762

NDCM: non-diabetic rats + contrast media, D: diabetic group, DCM: diabetic rats + contrast media, DR: diabetic rats + rosuvastatin, DRCM: diabetic rats + rosuvastatin + contrast media.

*P < 0.01 vs NDCM, D, DR and DRCM; # P < 0.05 vs D, DR, **P < 0.05 vs NDCM.

### Oxidative stress markers

As shown in Table 3, we had not found any difference between the DR group and the D group in terms of serum PCC, MDA, thiol and kidney TBARS levels. The kidney TBARS level was notably increased in DCM group than in D group and DR group (P < 0.01). Additionally, when the DCM group was compared to both the NDCM and DRCM groups, kidney TBARS was notably higher in the DCM group (P < 0.01). In group NDCM, kidney TBARS was notably increased than in groups D and DR (P < 0.05). Serum MDA was notably increased in group DCM than in groups D and DR (P < 0.01). The serum MDA level was notably lower in the DRCM group than in the DCM group (P < 0.01), but it was still higher than the levels in both group D and group DR (P < 0.05) (Table 3). In group NDCM, kidney TBARS was notably increased than in groups D, DR and DRCM (P < 0.05). When the DCM group was compared with group D and group DR, serum thiol levels were notably decreased in the DCM group (P < 0.01). However, in the DRCM group, serum thiol levels were notably higher than DCM group (P < 0.05). In group NDCM, levels of serum thiol were notably increased than in groups D, DR and DRCM (P < 0.05). The serum PCC level was notably increased in DCM group than in D group and DR group (P < 0.01). In the DRCM group, serum PCC levels were notably lower compared to the DCM group (P < 0.01). In group NDCM, levels of serum PCC were notably higher than in groups D, DR and DRCM (P < 0.05) and decreased than in group DCM (P < 0.01).

**Table 3:** **Oxidative stress markers of the study groups (mean ± SD)**

	**NDCM (n = 8)**	**D (n = 8)**	**DR (n = 8)**	**DCM (n = 8)**	**DRCM (n = 8)**
Kidney TBARS (nmol/g)	36.21 ± 5.21#	30.23 ± 6.30	29.38 ± 5.38	42.62 ± 6.12*	32.26 ± 4.28
Serum MDA (μmol/l)	7.23 ± 1.12#	3.03 ± 0.52	3.18 ± 0.61	9.25 ± 1.23*	4.56 ± 0.78+
Serum Thiol (μmol/l)	287.78 ± 29.23#	362.25 ± 58.16	343.16 ± 62.11	247.16 ± 23.87*	346.19 ± 30.18**
Serum PCC (μmol/l)	10.11 ± 1.34#	7.68 ± 1.21	8.12 ± 0.92	14.23 ± 1.73*	8.38 ± 1.19**

NDCM: non-diabetic rats + contrast media, D: diabetic group, DCM: diabetic rats + contrast media, DR: diabetic rats + rosuvastatin, DRCM: diabetic rats + rosuvastatin + contrast media.

*P < 0.01 vs NDCM, D, DR and DRCM; + P < 0.05 vs NDCM, D and DR; # P < 0.05 vs D, DR; **P < 0.05 vs NDCM.

### Inflammatory parameters

As shown in Table 4, there was not any significant difference between the DR group and D group in terms of serum IL-6 and TNF-α levels. When compared with either the D or DR group, the DCM group had higher serum levels of both IL-6 (P < 0.01) and TNF-α (P < 0.01). However, when compared to the DCM group, treatment with rosuvastatin reduced the CM-induced increase in both serum IL-6 (P < 0.05) and TNF-α levels (P < 0.05). In group NDCM, serum IL-6 and TNF-α levels were notably lower than in group DCM (P < 0.05).

**Table 4:** **Inflammatory parameters in the study groups (mean ± SD)**

	**NDCM (n = 8)**	**D (n = 8)**	**DR (n = 8)**	**DCM (n = 8)**	**DRCM (n = 8)**
IL-6 (pg/ml)	11.28 ± 1.45#	7.63 ± 0.93	7.23 ± 0.69	15.14 ± 1.23*	12.23 ± 1.06#
TNF-ɑ (pg/ml)	36.35 ± 5.67#	27.12 ± 2.31	25.34 ± 2.15	48.56 ± 5.23*	37.25 ± 4.12#

NDCM: non-diabetic rats + contrast media, D: diabetic group, DCM: diabetic rats + contrast media, DR: diabetic rats + rosuvastatin, DRCM: diabetic rats + rosuvastatin + contrast media.

*P < 0.01 vs NDCM, D, DR and DRCM; # P < 0.05 vs D and DR.

### Renal histopathology

**Figure 1:**
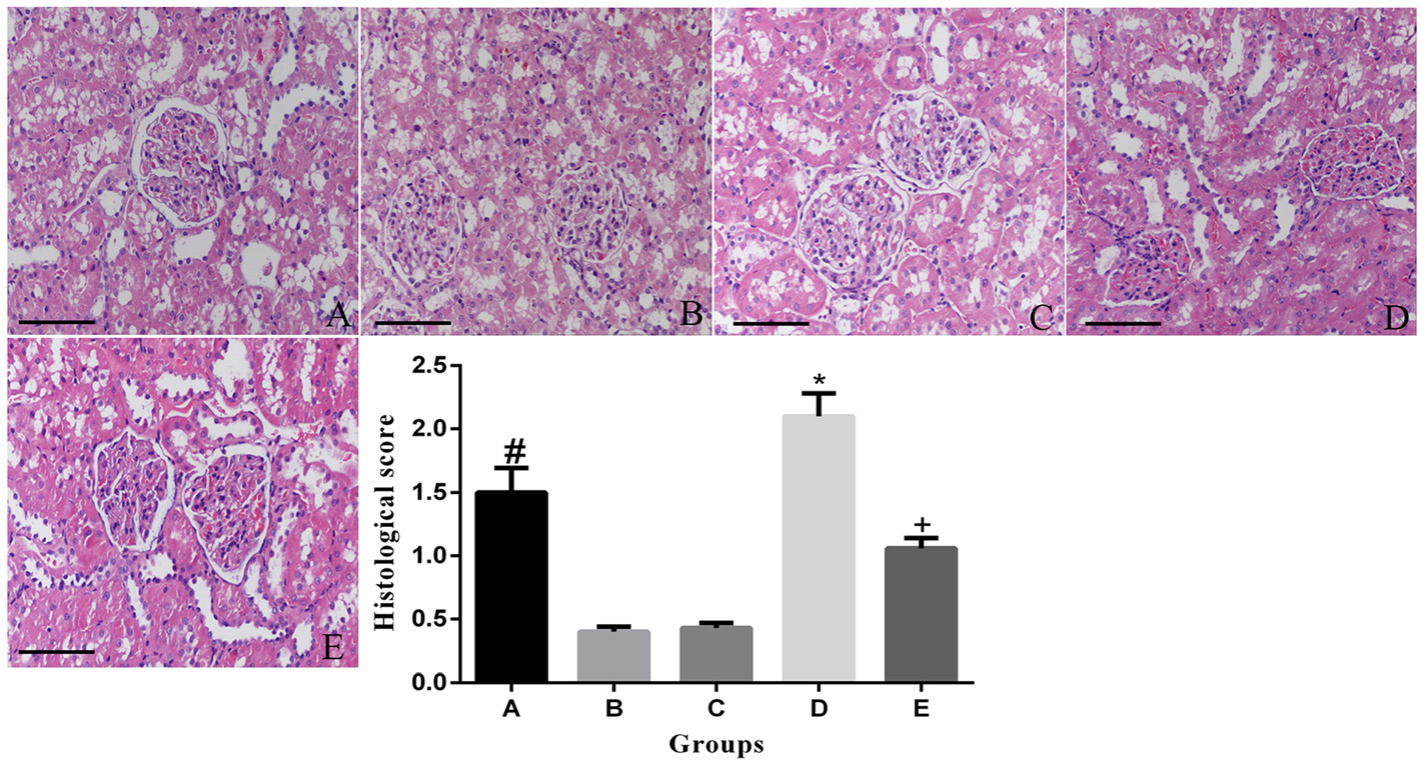
**Light microscopy of renal tissue from rats stained with hematoxylin and eosin. A**: non-diabetic rats + contrast media group, **B**: diabetic group, **C**: diabetic + contrast media group, **D**: diabetic + rosuvastatin group, **E**: diabetic + rosuvastatin + contrast media group. *P < 0.01 vs A, B, D and E; +P < 0.05 vs A, B and D; # P < 0.05 vs B and D. Scale bar: 50 μm.

As shown in Figure 1, in group NDCM, histological scores were notably decreased than in group DCM (P < 0.01). However, injury was found to be more severe in than DCM group than in groups D and DR (P < 0.01), but the degree of injury was less severe in DRCM group, compared with DCM group (P < 0.05), suggesting that rosuvastatin therapy significantly protected the kidney from the contrast media.

### Apoptosis

**Figure 2:**
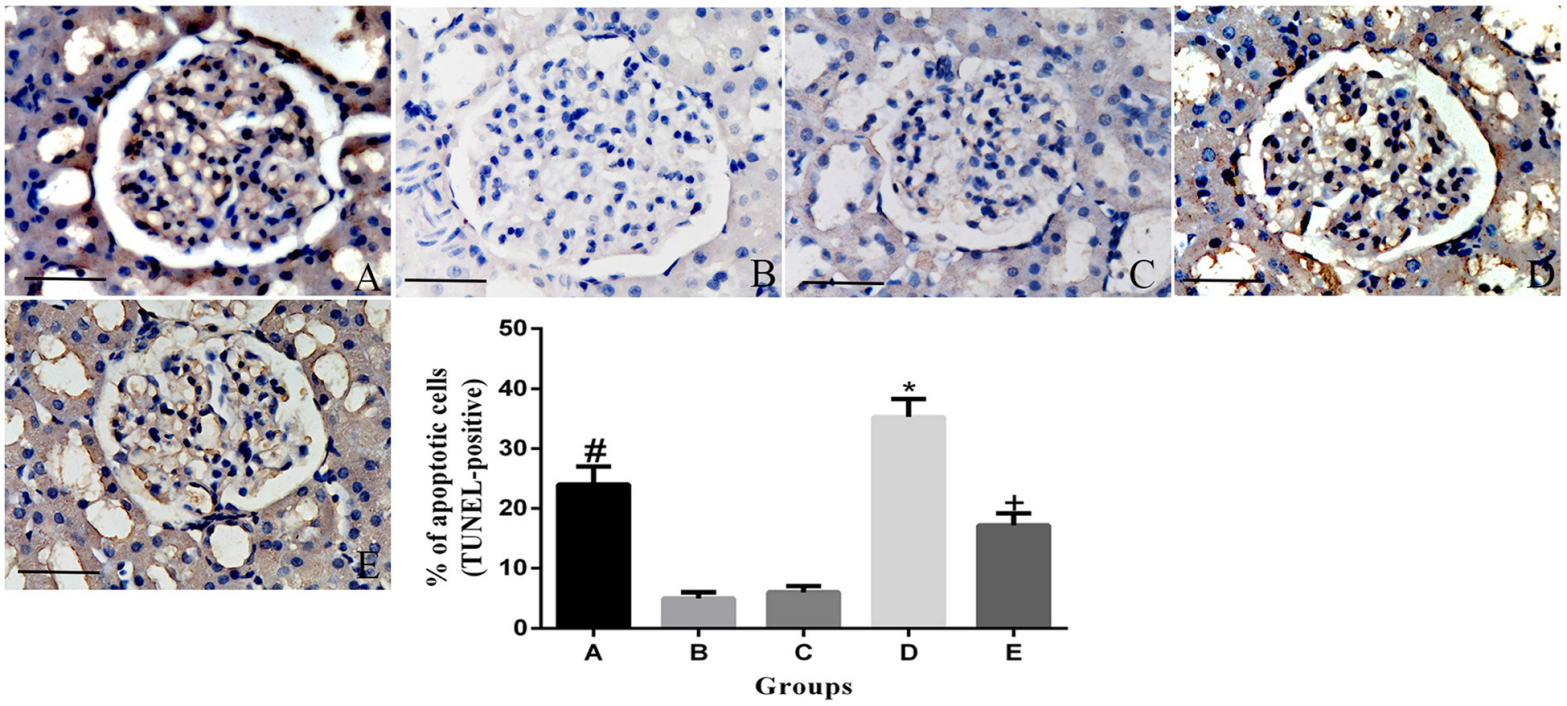
**Rosuvastatin inhibited contrast media-induced renal cell apoptosis.** Contrast media increases TUNEL-positive renal tubular cell number, whereas rosuvastatin pretreatment prevented contrast media-induced apoptosis. **A**: non-diabetic rats + contrast media group, **B**: diabetic group, **C**: diabetic + contrast media group, **D**: diabetic + rosuvastatin group, **E**: diabetic + rosuvastatin + contrast media group. *P < 0.01 vs A, B, D and E; +P < 0.05 vs A, B and D; # P < 0.05 vs B, D. Scale bar: 20 μm.

As shown in Figure 2, TUNEL staining showed that DCM showed a more significant extent compared with either the D group or DR group. The percentage of apoptotic cells was lower in the DRCM group than in the DCM group (P < 0.01). Additionally, the percentage of apoptotic cells was significantly higher in the DCM group than in the NDCM group (P < 0.01).

**Figure 3:**
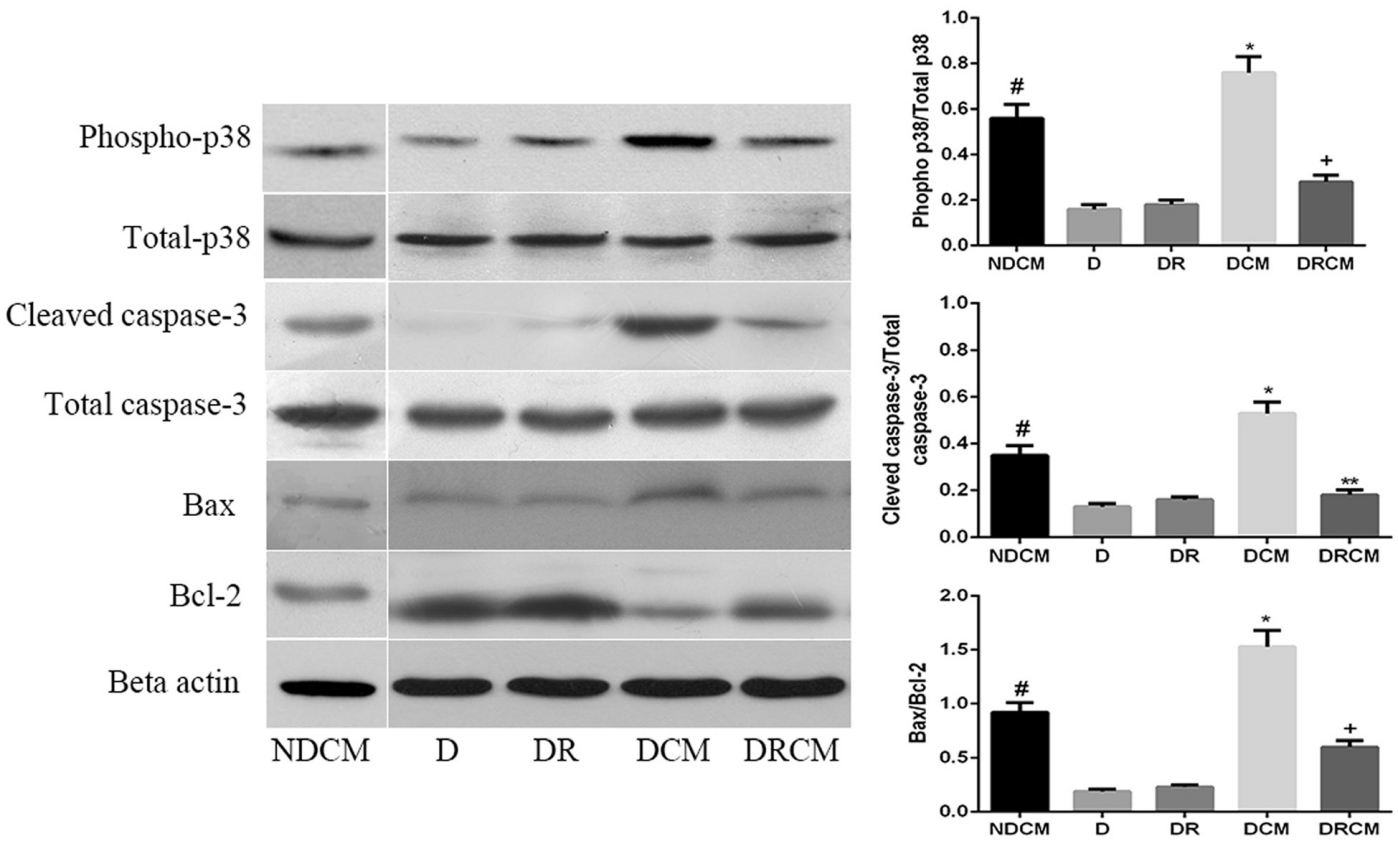
**Rosuvastatin decreased phospho-p38, cleaved caspase-3 and Bax/Bcl-2 protein expression by Western blotting.** Beta-Actin was used as a loading control. NDCM: non-diabetic rats + contrast media, D: diabetic group, DCM: diabetic + contrast media group, DR: diabetic + rosuvastatin group, DRCM: diabetic + rosuvastatin + contrast media group. *P < 0.01 vs NDCM, D, DR and DRCM; +P < 0.05 vs NDCM, D and DR; # P < 0.05 vs D and DR.

Previous studies have suggested that contrast media-induced apoptosis is mediated by activation of the p38 mitogen-activated protein kinase (MAPK) signal pathway [26]. As shown in Figure 3, the protein expression of phospho-p38 was notably increased in group DCM compared to groups D and DR (P < 0.01), and it was also higher in group DCM compared to group RCM (P < 0.01). In NDCM group, the protein expression of phospho-p38 was notably decreased compared with the DCM group. To confirm the presence of apoptosis in CIN rats, we investigated the caspase-3 substrate, Bax and Bcl-2 using Western blotting. The activation of caspase-3 was notably increased in DCM group compared to the NDCM, D and DR groups (P < 0.01). Additionally the activation of caspase-3 levels was notably reduced in group DRCM than in the DCM group (P < 0.01). The expression of the Bax/Bcl-2 ratio was notably increased in the DCM group than in D group and DR group (P < 0.01). When the DCM group was compared with the NDCM and DRCM groups, the Bax/Bcl-2 ratio was notably increased in DCM group (P < 0.01). In group NDCM, the expression of the Bax/Bcl-2 ratio was notably increased than in groups D and DR (P < 0.05).

## Discussion

For the first time, we investigated the effects of rosuvastatin treatment, initiated 5 days before the administration of contrast media to induce CIN in diabetic rats. We demonstrated that rosuvastatin decreased oxidative damage, inflammatory responses, kidney injury and apoptosis. Our findings support that rosuvastatin decreased CIN in diabetic rats.

In recent years, statins have been found to reduce the risk of CIN in DM patients [27]. Rosuvastatin has potent anti-inflammatory properties: it increases nitric oxide synthetase bioavailability, and reduces oxidative stress independent of its cholesterol-lowering effect [14]. We recently reported that rosuvastatin significantly decreases the CIN risk in DM patients with CKD undergoing arterial contrast media injection [18]. In current study, we found that there was a significant increase in renal injury biomarkers, including serum creatinine and creatinine clearance, in the rats administration with contrast media. Pretreatment with rosuvastatin significantly reversed the effects of the contrast media on serum renal injury markers, as well as creatinine clearance.

Contrast media induces a decrease in the expression of NO [16,22]. Rosuvastatin and other statins improve endothelial function by increasing NO synthetase bioavailability and decreasing oxidative stress [14]. In current study, we found that a decrease in product of nitrite, were the cause of elevation in creatinine levels and of renal histopathological alteration. We also found that levels of kidney nitrite were notably increased in DRCM group compared with DCM group after treatment with rosuvastatin. This may explain why renal injury was less significant in group DRCM than in DCM.

Our results showed that pretreatment with rosuvastatin reversed CM-induced oxidative stress in rats. CIN pathophysiology research shows that this condition is most same with result of renal ischemia, oxidative injury and direct toxicity to tubular epithelial cells [5,28]. After using contrast media, reactive oxygen species cause and enhance lipid peroxidation and cytotoxic damage, suggesting that oxidative injury is a major risk factor in the CIN pathogenesis [29]. A body of evidence has demonstrated that rosuvastatin has distinctive anti-inflammatory properties, improving oxidative injury and reducing oxidant and free radical generation [14,30-33]. We speculated that rosuvastatin decreases the contrast-induced explosion of serum MDA, PCC and renal TBARS and increases serum thiol levels, therefore ameliorating the renal histopathological findings in DRCM group.

Although the pathogenesis of CIN is still not completely understood, there is mounting evidence to support the critical role of inflammation in the pathogenesis of CIN [28,34]. TNF-α is a cascade-inducing pro-inflammatory cytokine that further recruits numerous mediators associated with endothelial and tissue injury. In the mouse model of nephrotoxicity, TNF-α was proven to play a central role in the activation of the inflammatory cytokine response [35]. IL-6 elicits many biological effects, one of which is the initiation of the inflammatory response [36]. It is synthesized in response to proinflammatory signals, and its production is also a common response to tissue injury and organ failure [37]. Contrast media treatment was followed by an increase levels of IL-6 in the kidney [38]. IL-6 and TNF-α are associated with CIN risk and poor long-term renal outcome after PCI [35,39]. Many Studies have showed that rosuvastatin may be as a therapeutic option in renal diseases that are characterized by preventing inflammation, apoptosis and fibrosis [40-43]. Our results showed that treatment with rosuvastatin reduced expression of serum IL-6 and TNF-α. Therefor rosuvastatin likely protects the kidney from CIN through the inhibition of renal inflammation. P 38 are also capable to enhanced IL-6 and TNF-α expression [35], our result confirm previous finding, and that rosuvastatin was able to prevent this effect.

Previous studies demonstrated that intrinsic or mitochondrial pathway involve in CM induces renal cells apoptosis [44]. In current study, we found that treatment with rosuvastatin prevented (1) CM-mediated caspase-3 activation, (2) CM-induced increase of ratio of Bax/Bcl-2, (3) CM-induced an increase in TUNEL staining cells, indicating that rosuvastatin prevents apoptosis in CIN. Other works have suggested that contrast media-induced apoptosis can be mediated by activation of the p38 MAPK in vitro and in vivo [26,45]. Current study showed that the rosuvastatin reduce CIN by suppression of apoptotic may be through inhibition of p38 MAPK pathways.

## Conclusions

Rosuvastatin attenuates contrast media-induced nephropathy by modulation of nitric oxide, inflammatory responses, oxidative stress and apoptotic processes, including p38 MAPK pathways. Our findings show that rosuvastatin may have an important role in preventing CIN.

## Abbreviations

CIN: Contrast-induced nephropathy
TBARS: Kidney thiobarbituric acid-reacting substances
MDA: Malondialdehyde
PCC: Protein carbonyl content
DM: Diabetes mellitus
LDL-C: Low-density lipoprotein cholesterol
FENa: Fractional excretion of sodium
IL-6: Interleukin-6
TNF-ɑ: Tumor necrosis factor-alpha
ELISA: Enzyme-linked immunosorbent assay
NO: Nitric oxide
MAPK: Mitogen-activated protein kinase
